# From root to shoot: quantifying nematode tolerance in *Arabidopsis thaliana* by high-throughput phenotyping of plant development

**DOI:** 10.1093/jxb/erad266

**Published:** 2023-07-11

**Authors:** Jaap-Jan Willig, Devon Sonneveld, Joris J M van Steenbrugge, Laurens Deurhof, Casper C van Schaik, Misghina G Teklu, Aska Goverse, Jose L Lozano-Torres, Geert Smant, Mark G Sterken

**Affiliations:** Laboratory of Nematology, Wageningen University & Research, 6708PB Wageningen, The Netherlands; Laboratory of Nematology, Wageningen University & Research, 6708PB Wageningen, The Netherlands; Laboratory of Nematology, Wageningen University & Research, 6708PB Wageningen, The Netherlands; Laboratory of Phytopathology, Wageningen University & Research, 6708PB Wageningen, The Netherlands; Laboratory of Nematology, Wageningen University & Research, 6708PB Wageningen, The Netherlands; Agrosystems Research, Wageningen University & Research, 6708PB Wageningen, The Netherlands; Laboratory of Nematology, Wageningen University & Research, 6708PB Wageningen, The Netherlands; Laboratory of Nematology, Wageningen University & Research, 6708PB Wageningen, The Netherlands; Laboratory of Nematology, Wageningen University & Research, 6708PB Wageningen, The Netherlands; Laboratory of Nematology, Wageningen University & Research, 6708PB Wageningen, The Netherlands; Ikerbasque, Spain

**Keywords:** *Arabidopsis thaliana*, biotic stress, growth rate analysis, *Heterodera schachtii*, high-throughput phenotyping, *Meloidogyne incognita*, root-parasitic nematodes, tolerance

## Abstract

Nematode migration, feeding site formation, withdrawal of plant assimilates, and activation of plant defence responses have a significant impact on plant growth and development. Plants display intraspecific variation in tolerance limits for root-feeding nematodes. Although disease tolerance has been recognized as a distinct trait in biotic interactions of mainly crops, we lack mechanistic insights. Progress is hampered by difficulties in quantification and laborious screening methods. We turned to the model plant *Arabidopsis thaliana*, since it offers extensive resources to study the molecular and cellular mechanisms underlying nematode–plant interactions. Through imaging of tolerance-related parameters, the green canopy area was identified as an accessible and robust measure for assessing damage due to cyst nematode infection. Subsequently, a high-throughput phenotyping platform simultaneously measuring the green canopy area growth of 960 *A. thaliana* plants was developed. This platform can accurately measure cyst nematode and root-knot nematode tolerance limits in *A. thaliana* through classical modelling approaches. Furthermore, real-time monitoring provided data for a novel view of tolerance, identifying a compensatory growth response. These findings show that our phenotyping platform will enable a new mechanistic understanding of tolerance to below-ground biotic stress.

## Teaser

The mechanisms of tolerance to root-parasitic nematodes remain unknown. We developed a high-throughput phenotyping system that enables unravelling of the underlying mechanisms of tolerance to nematodes.

## Introduction

The impact of root-parasitic nematodes on plant growth and development leads to reduced yield of food crops globally (Jones *et al.*, 2013; Bebber *et al.*, 2014). Interestingly, plants show intraspecific variation in their growth responses to below-ground biotic stresses by nematodes (Miltner *et al.*, 1991; Potter and Dale, 1994). In other words, plants display different disease burdens in response to similar initial nematode densities. The first measurable repression in plant growth (i.e. yield, plant height) by pathogen infection is defined as the tolerance limit (Seinhorst, 1986). The tolerance limit of plants is measured independently from resistance because tolerance and resistance are genetically and physiologically independent traits (Evans and Haydock, 1990; Teklu *et al.*, 2022). The tolerance limit is dependent on the plant and the nematode species, and the interaction between those two. Even though nematode tolerance has been known as a phenomenon for many decades, we lack an understanding of the underlying molecular and/or genetic mechanisms. Difficulties in quantifying tolerance and the required laborious screening methods hamper progress in this research area. Therefore, there is a need for a genetically tractable model species and a scalable screening method to better understand tolerance.

The model plant species should be able to support infections of the families of cyst nematodes (*Heteroderinae*) and root-knot nematodes (*Meloidogyninae*), as these belong to the most damaging obligate sedentary root parasites worldwide (Jones *et al.*, 2013; Quist *et al.*, 2015). Both genera spend most of their life cycle within roots. Cyst nematodes and root-knot nematodes cause significant damage to the root tissue by mechanical damage, large morphological changes, and loss of plant assimilates (Kyndt *et al.*, 2013). Infective second-stage juveniles (J2s) of cyst and root-knot nematodes invade the plant root and move through different tissue layers to reach the vascular cylinder (Wyss and Zunke, 1986; Wyss *et al.*, 1992). Cyst nematodes move brutally through cells, causing significant mechanical damage, whereas root-knot nematodes move in between cells by gently pushing them aside. After reaching the vascular cylinder, both cyst and root-knot nematodes induce a redifferentiation process that leads to the formation of multinucleated feeding cells known as syncytia and giant cells, respectively. The feeding cells are hypermetabolic sink tissues and serve as the sole source of nutrients for growing nematodes throughout their entire life cycle. This leads to fewer nutrients available for plant growth and development. Both nematode families consist of multiple species, of which the beet cyst nematode *Heterodera schachtii* and the root-knot nematode *Meloidogyne incognita* are the best studied, and therefore considered to be model species for plant–nematode interaction studies.

Up to now, tolerance to root-parasitic nematodes has been mainly studied in field crops (Seinhorst, 1986; Norshie *et al.*, 2011; Sasanelli *et al.*, 2013; Been *et al.*, 2015; Moosavi, 2015), making it difficult to resolve the underlying mechanisms as to why plants differ in their tolerance limits. Knowledge of the genetic underpinnings of tolerance is important for understanding how plants differ in their tolerance limits to root-feeding nematodes. The model plant *Arabidopsis thaliana* is a rich and often-used resource to study the role of natural genetic variation, and molecular and cellular mechanisms underlying nematode–plant interactions (Grunewald *et al.*, 2009; Absmanner *et al.*, 2013; Lozano-Torres *et al.*, 2014; Siddique *et al.*, 2014; Anwer *et al.*, 2018; Warmerdam *et al.*, 2018; Olmo *et al.*, 2020). It has been shown that *A. thaliana* harbours significant natural genetic variation in susceptibility to the beet cyst nematode *H. schachtii* and the root-knot nematode *M. incognita in vitro* (Anwer *et al.*, 2018; Warmerdam *et al.*, 2018). For instance, a quantitative trait locus allele of *ATS40-3* that was identified with a genome-wide association study affects the sex ratio of *H. schachtii* in Arabidopsis (Anwer *et al.*, 2018). Recently, we also showed that the transcription factor TCP9 modulates tolerance to *H. schachtii* via reactive oxygen species-mediated processes (Willig *et al.*, 2022). However, it remains unknown if *A. thaliana* harbours significant genetic variation in tolerance to nematodes.

So far, the Seinhorst yield loss model (SYLM) has mostly been used to determine the tolerance limit for plant-parasitic nematodes (Seinhorst, 1986; Norshie *et al.*, 2011; Sasanelli *et al.*, 2013; Been *et al.*, 2015; Moosavi, 2015). The SYLM models the density–yield relationship by an inverted sigmoid curve where loss of yield at a given time point is a derivative function of damage. Yield remains stable up to a certain nematode density (i.e. the tolerance limit) then declines exponentially, to end at a minimum yield level at the highest nematode density. The SYLM enables a comparison of tolerance limits of different crop varieties for a specific pathogen (Seinhorst, 1986). Importantly, herein, yield loss is dependent on the number of parasitic nematodes in the soil and not the number of successful infections detected at harvest (Seinhorst, 1986). The tolerance limit and the steepness of the decline in yield over increasing nematode densities are both agronomically important traits. Alternatively, for other pathogens, the slope of a regression of the selected host parameter against the pathogen load is made to determine if one plant is more tolerant than another (Pagán and Garcia-Arenal, 2020). However, neither linear regression nor the SYLM account for biological responses to pathogen infection such as overcompensation or compensation (i.e. increased growth speed or growth speed recovery) (Pedigo *et al.*, 1986).

Most of the studies that measure the effect of nematode infection on crop yield rely on destructive end point measurements (i.e. crop yield; Sasanelli *et al.*, 2013; Been *et al.*, 2015), leaving an information gap between the start and end point of the experiment. A few time-series experiments were performed by measuring the effect of soybean cyst nematode *Heterodera glycines* on the root system using a rhizotron (Miltner *et al.*, 1991) or by measuring the effect of *Meloidogyne chitwoodi* densities on the height of potato plants (Norshie *et al.*, 2011). Additionally, time-series measurements were also performed on the green canopy area upon nematode infection using a phenotyping design based on microplots (Joalland *et al.*, 2016, 2017). However, this latter design was limited to 70 microplots, which does not accommodate large phenotyping experiments including hundreds of genotypes for multi-genotype comparisons (i.e. genome-wide association studies or mutant screens). A system that is able to monitor a larger number of individually measured plants over time could provide insight into genes that modulate tolerance to root-parasitic nematodes.

Another recurring hurdle in tolerance research is to determine which plant trait should be measured. Most of the studies on tolerance to root-parasitic nematodes in potato focus on yield reduction. For example potato yield expressed in tuber weight and quality (Been *et al.*, 2015; Teklu *et al.*, 2022), which depend on a single destructive end point measurement. Others also measure the effect of nematodes on foliage or plant height which are not *per se* destructive (Norshie *et al.*, 2011), but these types of measurements are done less often because they are laborious. In *A. thaliana*, the green canopy growth, inflorescences, and the number of viable seeds can be used to measure tolerance to viruses (Shukla *et al.*, 2018). Most of the screening methods are laborious and/or time-consuming. For many purposes, these kinds of measurements might be adequate, but for large-scale phenotyping of multiple plant genotypes these laborious measurements hamper progress. Therefore, the chosen trait should also be easy to measure. However, it remains unknown which plant trait provides the most robust data for a high-throughput design to quantify tolerance to root-parasitic nematodes in *A. thaliana*.

Here we report on the construction and validation of a high-throughput phenotyping platform to monitor in real-time plant growth under below-ground biotic stress caused by nematode infection. Initially, we tested growth parameters (e.g. root system architectural components, green canopy area, flowering, and silique formation) to robustly quantify the effect of *H. schachtii* infestation on the growth and development of the host. We found that the green canopy area of Arabidopsis is an accessible and informative trait for automated analysis of growth responses to root-parasitic nematodes. Time-series measurements showed that daily growth rates of individual plants are congruent over plants and provided insight into biological responses of the host (i.e. compensation responses) that could not be determined in previous experimental designs. Therefore, we believe that our phenotyping system will not only allow for more accurate mechanistic studies into tolerance, but can also ultimately pinpoint genes that modulate tolerance to biotic stress by endoparasitic nematodes.

## Materials and methods

### Plant culturing

For seed sterilization, Arabidopsis seeds (Col-0, N60,000) were placed in Eppendorf tubes in a desiccator. The seeds were vapour sterilized for 3–4 h using a mixture of hydrochloric acid (25%) and sodium hypochlorite (NaOCl; 50 g l^–1^). Finally, the sterile seeds were stratified for 4 d. For *in vitro* assays, seeds were sown on square Petri dishes (120 × 120 mm) containing modified Knop medium (Sijmons *et al.*, 1991). For *in vivo* pot experiments, non-sterilized seeds were also stratified for 4 d and sown directly on top of silver sand. Seedlings were grown at 21 °C and 16 h light/8 h dark conditions. Seedlings in our high-throughput platform were grown at 19 °C and 16 h light/8 h dark conditions with LED lights (150 lumen).

### Hatching and sterilization of *Heterodera schachtii* and *Meloidogyne incognita*

The *H. schachtii* cysts (Woensdrecht population from IRS, the Netherlands) were separated from sand and roots of infected *Brassica oleracea* plants as previously described (Baum *et al.*, 2000) and were transferred into a clean Erlenmeyer flask containing water with 0.02% sodium (NaN_3_). This mixture was gently stirred for 20 min. Later, NaN_3_ was removed by vigorously washing with tap water. Cysts were then placed on a hatching sieve in a glass Petri dish. An antibiotic solution was added containing 1.5 mg ml^–1^ gentamycin, 0.05 mg ml^–1^ nystatin, and 3 mM zinc chloride. The cysts were incubated in the dark for 4–7 d. Thereafter, nematode juveniles were collected in a 2 ml Eppendorf tube. For *in vitro* experiments, J2s were surface-sterilized using a HgCl_2_-containing solution (0.002% Triton X-100 w/v, 0.004% NaN_3_ w/v, 0.004% HgCl_2_ w/v) for 20 min. After incubation, the nematodes were collected by centrifugation and the supernatant was removed. Nematodes were washed with sterile water and spun down again. This was repeated three times. Finally, for *in vitro* experiments, the nematodes were resuspended in 0.7% gelrite (Duchefa Biochemie, Haarlem, the Netherlands). For *in vivo* experiments, hatched J2s were separated from debris by centrifugation in a 70% sucrose gradient. Afterwards, the nematodes were washed by centrifugation in tap water three times. Finally, the nematodes were resuspended in tap water.

Eggs of *M. incognita* were obtained by soaking *M. incognita* (strain ‘Morelos’ from INRA, Sophia Antipolis, France) with 0.05% (v/v) NaOCl for 3 min. Roots were rinsed with tap water and the eggs were collected on a 25 µM sieve. Next, the eggs were incubated in a solution of 2.4 mM NaN_3_ for 20 min while swirling. Thereafter, the eggs were rinsed with tap water and incubated on a 25 µM sieve in a solution of 1.5 mg ml^–1^ gentamycin, 0.05 mg ml^–1^ nystatin in the dark at room temperature. After 4 d, hatched juveniles were collected. For *in vivo* experiments, hatched J2s were separated from debris by centrifugation in a 70% sucrose gradient. Afterwards, the nematodes were washed by centrifugation in tap water three times. Finally, the nematodes were resuspended in tap water.

### Quantifying the root system architecture of nematode-infected Arabidopsis

Nine-day-old *in vitro* grown *A. thaliana* seedlings were individually inoculated with increasing densities (*P*_i_) of surface-sterilized J2s of *H. schachtii* (0–50 juveniles ml^–1^ of modified KNOP medium). Roots of nematode-infected plants were scanned at 7 days post-inoculation (dpi) using an Epson Perfection V800 photo scanner. Various root measurements were conducted (i.e. total root length, main root length, total secondary root length, and average secondary root length), collectively referred to as the root system architecture. Measurements were taken using the WinRHIZO package for Arabidopsis (WinRHIZO pro2015, Regent Instrument Inc., Quebec, Canada). The number of root tips was counted manually based on the scans made by WinRHIZO. Differences in the root length per seedling in centimetres and the number of root tips were statistically analysed with a one-way ANOVA with post-hoc Tukey’s HSD test in R.

### Quantifying above-ground development and growth of *Heterodera schachtii-*infected Arabidopsis plants

Three-week-old *A. thaliana* seedlings were inoculated with increasing densities of *H. schachtii* (0–50 juveniles g^–1^ of dry sand). During a period of 14 dpi, we recorded the green canopy area, the number of flowers, the number of siliques, and the number of basal stems every second day with a video camera. By using Adobe Photoshop (Version: 22.5.6 20220204.r.749 810e0a0 x64), we manually isolated the green canopy area from the images and analysed the area manually using ImageJ. Differences per treatment per time point were analysed using one-way ANOVA with post-hoc Tukey’s HSD test in R.

### High-throughput analysis of the green canopy area of nematode-infected Arabidopsis

Pots filled with silver sand were placed in stainless steel frames made (Supplementary Fig. S1A, D) and covered with a 3 mm thick black non-reflective foamed PVC coversheet (Supplementary Fig. S1B, C, E) drilled with countersunk holes ~73 mm apart. Extra holes were drilled with a diameter of 7 mm, and 4 mm away from the countersunk holes for nematode inoculations. Prior to sowing, silver sand was watered with Hyponex (1.7 mM NH4^+^, 4.1 mM K^+^, 2 mM Ca^2+^, 1.2 mM Mg^2+^, 4.3 mM NO_3_^–^, 3.3 mM SO_4_^2–^, 1.3 mM H_2_PO_4_^–^, 3.4 µM Mn, 4.7 µM Zn, B 14 µM, 6.9 µM Cu, 0.5 µM Mo, 21 µM Fe, pH 5.8) for 5 min. Seven days after sowing, seedlings were watered again for 5 min. Nine-day-old seedlings were inoculated with increasing densities of *H. schachtii* and *M. incognita* (0–100 juveniles g^–1^ of dry sand). Seedlings were inoculated by first punching a hole ~3.5 cm deep in the soil, into which 1 ml of tap water containing a measured number of nematodes was added using a pipette. For our experiments, we did not use a blocking design as it would greatly increase the chance for error when manually inoculating plants. Pictures of the Arabidopsis seedlings were taken every hour (15 pictures d^–1^) during the light period automatically for a period of 21 d with 12 UI-1490LE-C-HQ cameras (IDS Imaging) mounted with 12 mm lenses on the ceiling of the climate chamber (Cat. No. B5M12056, IDS Imaging). Because of the purple LED light, colour corrections were done using Adobe Photoshop (Version: 22.5.6 20220204.r.749 810e0a0 x64). The surface area of the rosette was determined using a custom-written ImageJ macro [ImageJ 1.51f; Java 1.8.0_321 (32 bit)] (Supplementary Protocol S1) and Java was used to make GIFs (Supplementary Protocol S2). In the GIFs and images of individual plants, a green artefact can be observed for all plants and images which is caused by algal growth. This error is systemic and occurs for all plants; however, after day 4, the impact on the green canopy area is minimal. Another point to note is that the number of measured plants varies between experiments due to technical issues (i.e. not enough nematodes), abnormal growth (clearly smaller than the general trend within inoculum densities), or plants that died due to extremely high densities of nematodes. More information about how the experiment was performed can be found in: doi: dx.doi.org/10.17504/protocols.io.kqdg39167g25/v1.

### Seinhorst yield loss model (SYLM)

To quantify the tolerance limit for *H. schachtii* infection in Arabidopsis, we fitted the green canopy area data to Seinhorst’s equation (Seinhorst, 1986) (Equation 1). Before fitting the model to the data, each measurement was averaged over the day and replicates for each nematode density:


$$\begin{array}{l} y=m+\left(1\;\;m\right)\times 0.95^{\left(P\text{i}/T_{\text{SYLM}}-1\right)}\text{ for }P_{\text{i}}>T_{\text{S}YLM}\\ y\;=\;1\text{ for }P_{\text{i}}<T_{SYLM} \end{array}$$ (1)

where *y* is the relative yield defined as the ratio between the canopy surface area and the non-inoculated canopy area surface area (control), *m* is the minimum relative yield, *P*_i_ is the initial nematode density in nematodes g^–1^ of soil, and *T*_SYLM_ is the tolerance limit (nematodes g^–1^ of soil). The assumption in the model (Seinhorst, 1986) is that below the tolerance limit, plant yield is equal to a situation where *P*_i_=0 (not infected).

Using these equations, the tolerance limit *T*_SYLM_ and the minimum yield *m* were estimated (model and code available via gitlab: https://git.wur.nl/published_papers/willig_2023_camera-setup).

### Plant growth analysis using the high-throughput phenotyping platform

To analyse the growth data of the plants obtained from the high-throughput platform, custom scripts and functions were written in ‘R’ (available via gitlab: https://git.wur.nl/published_papers/willig_2023_camera-setup). For analysis, we used the median daily leaf area (cm^2^), which was calculated by taking the median leaf area of the daily ­measurements (15 per day). The data were log_2_ transformed before analysis for ­normalization. The rate of growth was determined per day per plant by (Equation 2):


$$\begin{array}{l} R_{x,t}=\log_{2}\left(A_{x,\;\;\;\;\;\;\;t-1}-A_{x,\;\;\;\;\;\;\;t}\right) \end{array}$$ (2)

where *R*_*x,t*_ is the transformed growth rate of plant *x* at day *t* from day *t–*1 to day *t* based on the median green canopy area *A*_*x,t*_. Differences in growth rate were determined using a Wilcoxon Rank Sum test as implemented in the ggpubr package (https://cran.r-project.org/web/packages/ggpubr/index.html).

Differences in growth rate between plants infected with either *H. schachtii* or *M. incognita* were tested using a paired *t*-test comparing data from the same day and density combination. Data were multiple-testing corrected using the p.adjust function with the fdr method (Benjamini and Hochberg, 1995).

### Modelling growth rates

Growth models were fitted to the data using the growthrates package, which was used to fit a logistic growth model for the median green canopy area A_t_ (cm^2^) (Equation 3):


$$\begin{array}{l} A_{t}=\frac{K\;\;\;\;\;\;\;\times A_{0}}{A_{0}+\left(K-A_{0}\right)\times e^{\left(-r\times t\right)}} \end{array}$$ (3)

where the parameters *K* is the maximum green canopy area (cm^2^), *A*_0_ is the initial canopy area (cm^2^), and *r* is the intrinsic growth rate (d^–1^) determined as a function of time *t* (d). These fitted values were used to explore the relationship between nematode density and the *K* and *r*. Bad fits (*P*<0.05) were removed before analysis.

We found that the relationship between *K* and density could be described by the Gaussian function (Equation 4):


$$\begin{array}{l} K=K_{\text{B}}+\frac{K_{\;\;\;\sigma \;\;\;}}{P_{\;\;\;\sigma \;\;\;}}\times e^{-{\left(\frac{P_{\text{i}}-P_{\text{M}}}{P_{\;\;\;\sigma \;\;\;}}\right)}^{2}} \end{array}$$ (4)

where *P*_i_ is the initial nematode density in nematodes per g of soil, *K*_B_ is the basal canopy size, *K*_σ_ is the normalized maximum canopy area that can be achieved over the *P*_i_ range, *P*_σ_ is the deviation around the nematode density allowing maximum growth, and *P*_M_ is the nematode density at which maximum growth is achieved. Note that this allows for the maximum growth to be achieved at *P*_i_ ≥0, which is not possible in the SYLM, which assumes maximum growth at *P*_i_=0.

We found that the relationship between the intrinsic growth rate *r* and the initial nematode density *P*_i_ could be described by the exponential function (Equation 5):


$$\begin{array}{l} r=r_{\text{B}}+r_{\text{A}}\times e^{-\left(c_{P\text{i}}\times P_{\text{i}}\right)} \end{array}$$ (5)

where *r*_B_ is the basal growth rate (d^–1^), *r*_A_ is the adaptive growth rate (d^–1^), and *c*_*P*i_ is the translation constant (g of soil per nematode density), which translates the *P*_i_ to an impact on growth rate.

Together, the function for *K* and *r* could be used to model the time-series data of the entire experiment in the logistic growth model. We modelled the parameter values using nls and extracted confidence intervals using the nlstools package. In this model, the tolerance limit can be defined as 2×*P*_M_ (where the achieved canopy area is equal to the achieved canopy area at *P*_i_=0).

## Results

### Density–response relationship between plant architecture and nematode inoculation density

**Fig. 1.**
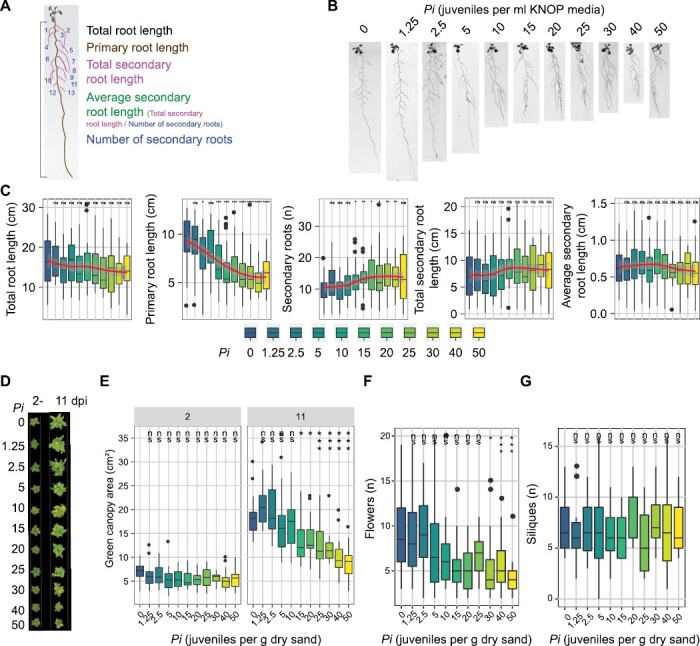
The primary root length and the green canopy area of Arabidopsis respond in a density-dependent manner to infection by *H. schachtii*. (A–C) Nine-day-old Arabidopsis seedlings were inoculated with increasing densities of *H. schachtii* juveniles (0–50 ml^–1^ of modified KNOP medium). The growth of the seedlings was monitored and measured at 7 dpi. (A) Overview of root system architecture components that we have quantified. (B) Representative images of the Arabidopsis root system at 7 dpi. (C) Total root length (cm), primary root length (cm), total secondary root length (cm), average secondary root length (cm), and the number of secondary roots (*n*). Data were analysed by one-way ANOVA with post-hoc Tukey HSD; ns=not significant, *P<0.05, **P<0.01, ***P<0.001 (*n*=24–30). (D–G) Twenty-one-day-old Arabidopsis seedlings cultivated in soil were inoculated with increasing densities of second juveniles of *H. schachtii* (0–50 juveniles g^–1^ of dry sand). (D) Representative images of Arabidopsis seedlings above-ground at 2 and 11 dpi. (E) Green canopy area (cm^2^) at 2 and 11 dpi with increasing densities (*P*_i_). (F) Number of flowers at 11 dpi. (G) Number of siliques at 11 dpi. Data were analysed by one-way ANOVA with post-hoc Tukey HSD; ns=not significant, *P<0.05, **P<0.01, ***P<0.001 (*n*=16–20).

The impact of biotic stresses on plant growth and development can be expressed as a function of inoculation density of causal agents of disease. To determine which plant growth parameters sensitively reflect the impact of nematode challenge on *A. thaliana*, we monitored changes in root system architecture and above-ground plant features at increasing inoculation densities of the beet cyst nematode *H. schachtii*. We first quantified different root system architecture components (i.e. primary root length, the number of secondary roots, and secondary root length) after inoculating *in vitro* cultured Arabidopsis plants with increasing densities (*P*_i_) of *H. schachtii* (Fig. 1A). At the system architecture level, we found that challenging Arabidopsis with *H. schachtii* at higher inoculation densities disturbs the typical regular patterning of emerging lateral roots (Fig. 1B). However, despite disturbing the lateral root patterning, the total root length did not change by increasing inoculation densities of *H. schachtii* (Fig. 1C). We did observe a decrease in primary root length around *P*_i_ ≥5, but this was compensated by an increase in the number of secondary roots (Fig. 1C).

Next, we monitored the impact of *H. schachtii* densities on above-ground plant growth and development of *A. thaliana* cultivated in soil by daily assessing changes in green canopy area, the number of flowers, and the number of siliques (Fig. 1D). The above-ground plant growth/development over time showed clear detrimental effects linked to increased *H. schachtii* infection pressure (Supplementary Fig. S2A). At 11 dpi, we observed the biggest differences in green canopy area between inoculation densities, including a tolerance limit of Arabidopsis for *H. schachtii* at 15 juveniles g^–1^ of soil (Fig. 1D, E). We also counted the number of flowers and siliques over time (Supplementary Fig. S2B, C). Likewise, we also observed a density-dependent response for the number of flowers, albeit at higher inoculation densities (Fig. 1F). In contrast, we did not find a significant response in the number of siliques by inoculation density (Fig. 1G). In conclusion, both primary root length and green canopy area can be used to assess the impact of nematode-induced biotic stress on *A. thaliana.*

We tested whether the green canopy area could also be used as a proxy for below-ground adaptations to nematode challenge, as repeated measurements on root system architecture require a more artificial experimental design. To address this question, we calculated the Spearman’s rank order correlation coefficient for our measurements of green canopy area and root system architecture components acquired through different experimental designs (Supplementary Fig. S3). Indeed, we found that green canopy area strongly correlates with primary root length (*R*^2^=0.89, *P*<0.0001) (Supplementary Fig. S3B). Green canopy area also anticorrelated well with the number of secondary roots (*R*^2^*=* −0.63, *P* = 0; Supplementary Fig. S3E) and total secondary root length (*R*^2^= −0.64, *P* = 0.04; Supplementary Fig. S3C). As expected, we found no significant correlation between the green canopy area and average secondary root length (Supplementary Fig. S3D) and total root length (Supplementary Fig. S3A). Based on this analysis, we focused further on developing a high-throughput automated phenotyping platform using the green canopy area of Arabidopsis cultivated in soil to assess the impact of *H. schachtii* at different inoculation densities over time.

### High-throughput monitoring of density–response relationships between nematode densities and green canopy area

**Fig. 2.**
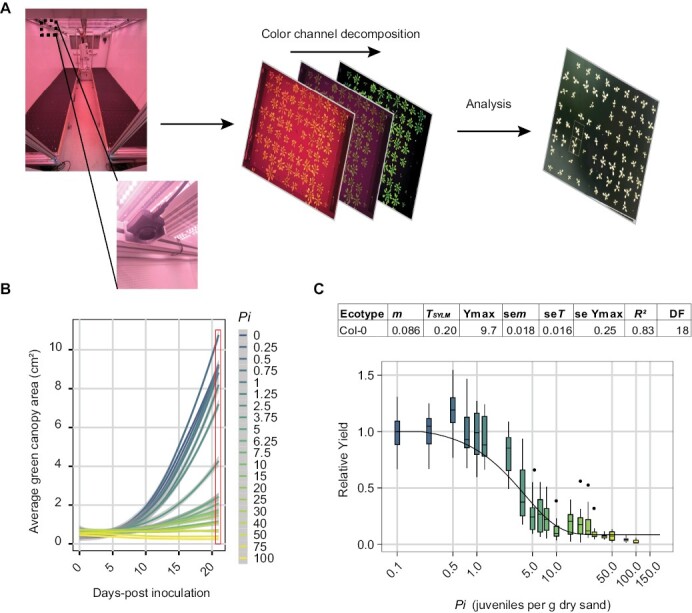
The relationship between the inoculation density (*P*_i_) of *H. schachtii* and green canopy area (relative yield). Nine-day-old Arabidopsis seedlings were inoculated with 20 different densities of *H. schachtii* juveniles (0–100 J2s g^–1^ of dry sand) in 200 ml pots containing 200 g of dry sand. (A) Experimental set up and colour channel decomposition of photos to extract the green leaf surface from the images. (B) Average growth curve of Arabidopsis plants inoculated with different inoculum densities of *H. schachtii* from 0 to 21 dpi. Line fitting was based on a LOESS regression. The red box indicates the data that are used for fitting the Seinhorst yield loss equation (*n*=10–24). (C) Fitting according to the Seinhorst yield loss equation. Parameter values for Seinhorst’s equation for the relationship between initial population density (*P*_i_) of *H. schachtii* and measured leaf surface area. *P*_i_ and tolerance limit (*T*_SYLM_) are expressed in *H. schachtii* (per g of dry sand) while, the minimal yield (*m*) is the lowest proportion of the maximum green canopy area (cm^2^) (*Y*_max_) at 21 dpi. The goodness of fit of the model on the data is expressed as the coefficient of determination (*R*^2^).

Our high-throughput automated monitoring platform can record and analyse changes in green canopy area of 960 Arabidopsis plants simultaneously for a period of 21 d (Fig. 2A). The platform design includes stainless steel frames that can hold 200 ml pots filled with soil (Supplementary Fig. S1). The frames fix the pots in position and give anchor to perforated matte black cover plates which maximize the contrast of the green canopy area. The frames and cover plates match the size of a flooding table placed in a fully automated climate room. Each frame contains 160 perforated holes to sow seeds and inoculate the pots. Photographs can be taken at any desired time interval by high-resolution cameras mounted at fixed positions above the cover plates. To monitor the impact of nematode challenge on green canopy area, photographs were taken at 1 h intervals for 21 d during the daytime (i.e. 15 pictures d^–1^). To collect data on individual plants, the pictures were processed by colour channel decomposition in Adobe Photoshop and analysed in ImageJ (Fig. 2A). Every experiment can result in 302 400 pictures of individual plants (315 pictures per plant).

**Fig. 3.**
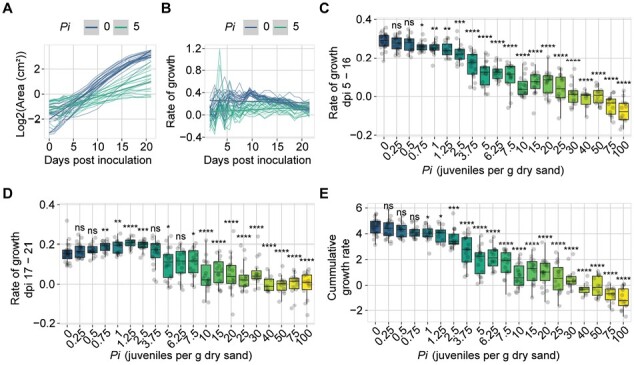
Quantification of the tolerance limit of Col-0 to *Heterodera schachtii* based on growth rate. Nine-day old Arabidopsis seedlings were inoculated with 20 different densities (*P*_i_) of *H. schachtii* juveniles (0–100 juveniles g^–1^ of dry sand). (A) Green canopy area of plants treated with *P*_i_*=*0 (blue line) or *P*_i_*=*5 (aquamarine line) from 0 to 21 dpi. (B) Rate of growth of plants treated with *P*_i_*=*0 (blue line) or *P*_i_*=*5 (aquamarine line) from 0 to 21 dpi was calculated using Equation 2. (C) Median rate of growth of plants inoculated with increasing *P*_i_ values growing from 5 to 16 dpi. (D) Rate of growth (cm^2^) of plants inoculated with increasing *P*_i_ values growing from 17 to 21 dpi. (E) Cumulative growth rate of plants inoculated with increasing *P*_i_ values. (C–E) Dots represent individual plants. Data were analysed with a Wilcoxon rank sum test; ns=not significant, *P<0.05, **P<0.01, ***P<0.001 (*n*=10–24).

To validate our system, we inoculated 480 *A. thaliana* Col-0 seedlings with increasing densities of *H. schachtii* (*P*_i_ 0–100 juveniles g^–1^ of dry sand) and monitored their growth for a period of 3 weeks. The green canopy area rendered from the recordings was collected and analysed (Supplementary Video S1; Fig. 2B). We confirmed that increasing densities of *H. schachtii* decreased the growth of Arabidopsis plants. To test if changes in the green canopy area fit the SYLM, we tested our green canopy area at 21 dpi for its fit (Equation 1) (Seinhorst, 1986). We found the typical inverted sigmoid curve, and also that our data fit well to the SYLM (*R*^2^=0.83; Fig. 2C), indicating that there is a strong relationship between nematode densities and the green canopy area. Also, we found that the tolerance limit (*T*_SYLM_) is placed around *P*_i_=0.21 (Fig. 3C). Altogether, we concluded that we have developed a high-throughput system in which we can quantify the relationship between green canopy size and cyst nematode inoculum densities using the Seinhorst equation for yield losses for Arabidopsis.

### Quantifying tolerance limits on growth rates

The SYLM does not accommodate for compensation effects we observed at low inoculation densities (Fig. 2C). Based on the boxplots, the tolerance limit is between *P*_i_ values of 1.25 and 2.5, whereas the SYLM placed it at 0.21. Another drawback of the SYLM is that data of a single time point of a series is tested for fit, ignoring changes over time. To take the dynamics in plant response into account, we sought an alternative way to quantify tolerance limits that uses all data collected over time (Fig. 2B). It is described that a delay in plant growth occurs at lower nematode densities and that higher nematode densities may lead to earlier cessation of growth (Seinhorst, 1986). Therefore, we extracted the growth rate (Equation 2) of plants inoculated with different nematode densities from 0 to 21 dpi to test if changes in growth rates can be used to determine tolerance limits.

First, we calculated the growth rate per day of individual plants that were inoculated with increasing *H. schachtii* densities (Equation 2) (Fig. 3A, B; Supplementary Figs S4–S6). Plants that were mock-inoculated showed a consistent growth rate in green canopy area between 5 and 15 dpi (Fig. 3C). Plants inoculated with *P*_i_ ≥0.75 had a lower growth rate compared with mock-treated plants (Fig. 3C). From 15 dpi onwards, growth rates of mock-inoculated plants reached a stationary phase, while plants inoculated with nematodes (*P*_i_*=*0.75–2.5) continued their exponential growth rate (Fig. 3D). Finally, we calculated the cumulative growth rate, which is the sum of all growth rates over time per plant (Fig. 3E), which showed that plants treated with *P*_i_ ≥1 grew significantly more slowly compared with mock-inoculated plants. Based on these results, we conclude that the tolerance limit of Arabidopsis Col-0 for *H. schachtii* is around *P*_i_*=*1.

### Quantifying tolerance limit to *M. incognita*

**Fig. 4.**
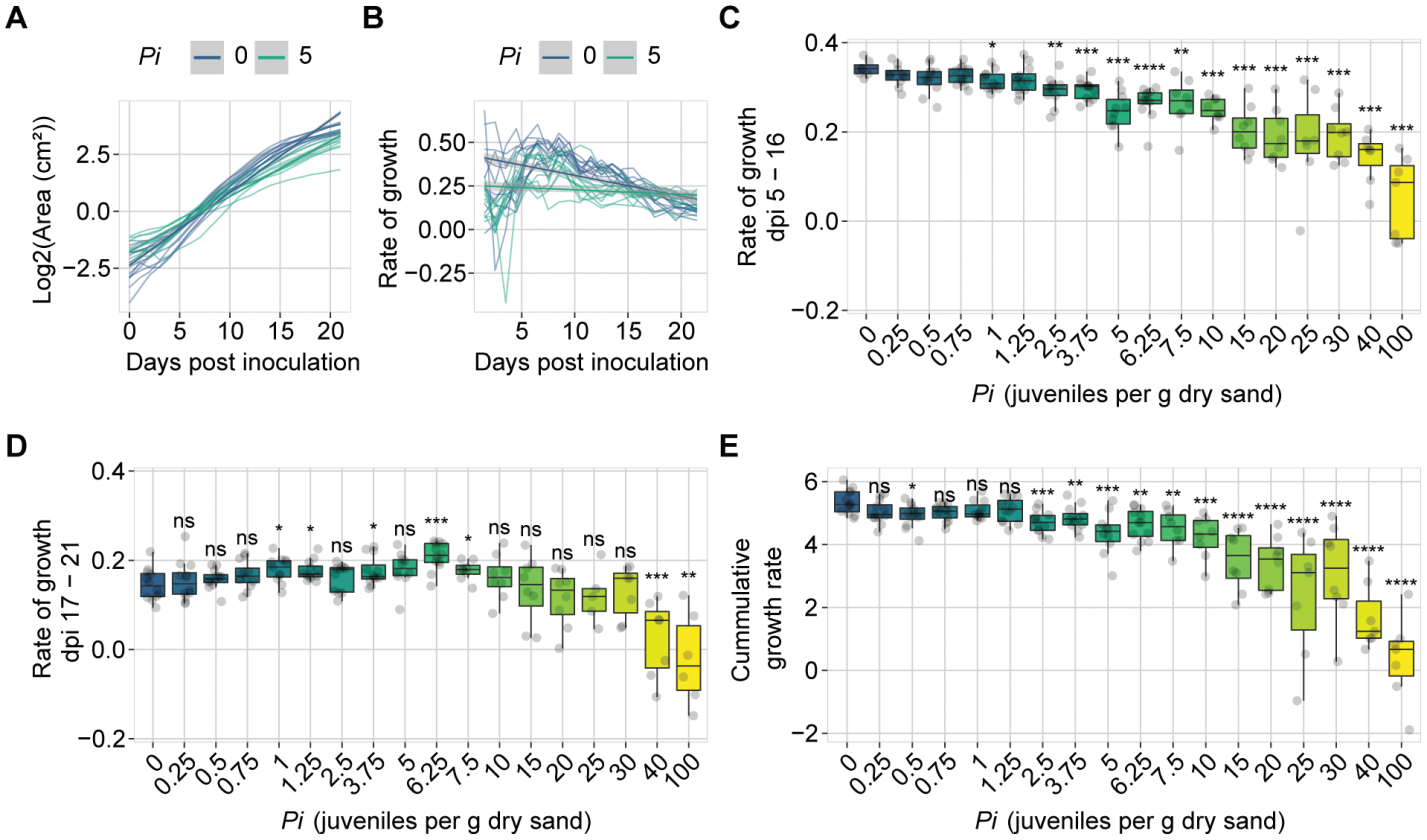
Quantification of the tolerance limit of Col-0 to *Meloidogyne incognita* based on growth rate. Nine-day-old Arabidopsis seedlings were inoculated with 18 different densities (*P*_i_) of *M. incognita* juveniles (0–100 juveniles g^–1^ of dry sand. (A) Green canopy area of plants treated with *P*_i_=0 (blue line) or *P*_i_*=*5 (aquamarine line) from 0 to 21 dpi. (B) Rate of growth of plants treated with *P*_i_*=*0 (blue line) or *P*_i_*=*5 (aquamarine line) from 0 to 21 dpi was calculated using Equation 2. (C) Rate of growth of plants inoculated with increasing *P*_i_ values growing from 5 to 16 dpi. (D) Rate of growth of plants inoculated with increasing *P*_i_ values growing from 17 to 21 dpi. (E) Cumulative growth rate of plants inoculated with increasing *P*_i_ values. (C–E) Dots represent individual plants. Data were analysed with a Wilcoxon rank sum test. ns=not significant, *P<0.05, **P<0.01, ***P<0.001 (*n*=10–12).

Root-knot nematodes migrate through the roots intercellularly and therefore cause less damage than cyst nematodes. Hence, we hypothesized that the tolerance limit for *M. incognita* infections should be at a higher *P*_i_ than for *H. schachtii*. To this end, 320 nine-day-old Arabidopsis seedlings were inoculated with 18 different densities (*P*_i_*=*0–100) of *M. incognita* and monitored for 21 d. We fitted the data to the SYLM and found a *T*_SYLM_ of 0.57 (Supplementary Fig. S7). As we observed for *H. schachtii*-infected plants, we found that the model underestimates the Tolerance limit. We thus also calculated the growth rates to capture the dynamics of the system (Fig. 4A–E; Supplementary Fig. S8). Here, we observed that the growth rates of plants between 5 and 16 dpi was less affected by *M. incognita* than by *H. schachtii* (Fig. 4C). The growth rates of plants treated with *P*_i_ ≥1.25 were significantly slower. From 17 to 21 dpi, we observed that plants inoculated with *M. incognita* at *P*_i_*=*1–7.5 tended to have a higher growth rate than mock-inoculated plants (Fig. 4D). Plants inoculated with *P*_i_*=*40 and 100 stopped growing altogether. Next, we calculated the cumulative growth rate, which showed that plants treated with *P*_i_ ≥2.5 grow significantly more slowly compared with mock-inoculated plants (Fig. 4E). Based on these results, we concluded that our high-throughput phenotyping system can also determine tolerance limits to root-knot nematodes and that the tolerance limit of *A. thaliana* Col-0 to *M. incognita* infection is around *P*_i_*=*2.5.

### Arabidopsis Col-0 is more tolerant to *M. incognita* than to *H. schachtii*

Next, we correlated the growth rates of *M. incognita*-inoculated plants to *H. schachtii*-inoculated plants per time point and per inoculation density to determine if the growth rates of *A. thaliana* Col-0 are less affected by initial nematode densities of *M. incognita* than by *H. schachtii* (Supplementary Fig. S9A, B). Both correlations based on time point and inoculum density between *H. schachtii*- and *M. incognita*-inoculated plants indicate that plants inoculated with *M. incognita* retain higher growth rates than plants inoculated with the same density of *H. schachtii*. Subsequently, we tested if there are significant differences between growth rates per *P*_i_ and per time point. For instance, we observed that *H. schachtii* infection has more impact on *A. thaliana* growth rates than *M. incognita* at *P*_i_*=*5, especially from 5 to 10 dpi (Supplementary Fig. S10A). From 11 to 21 dpi, the differences were smaller but often still significant. Then, we calculated the difference in growth rate by extracting the *H. schachtii*-treated growth rates from the *M. incognita*-treated growth rates per time point and *P*_i_ (Supplementary Fig. S10B). We found that most of the growth rates of plants inoculated with *M. incognita* are higher than those of *H. schachtii*-inoculated plants. Also, the largest differences in growth rates were visible from 5 to 10 dpi (Supplementary Fig. S10B, C).

**Fig. 5.**
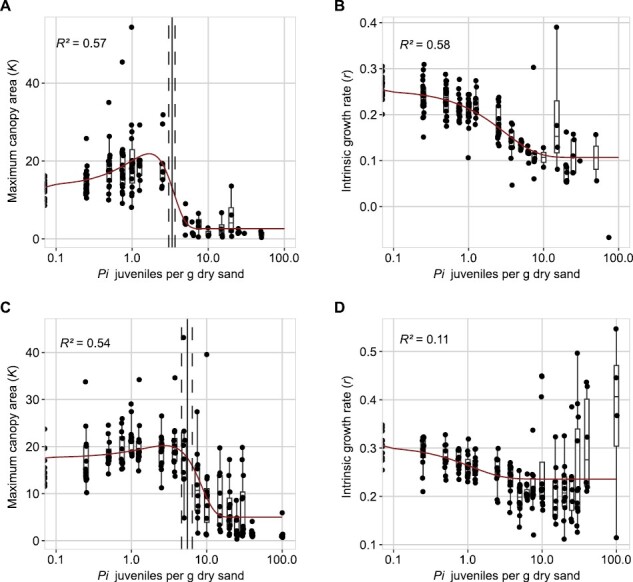
*Arabidopsis thaliana* Col-0 is more tolerant to *Meloidogyne incognita* than to *Heterodera schachtii*. Fitted logistic growth rate parameters of *A. thaliana* plants infected with *H. schachtii* or *M. incognita*. (A) The maximum canopy area *K* per infection density of *H. schachtii*. The fitted line is from a Gaussian curve, the *R*^2^ is from the fitted curve. (B) The intrinsic growth rate *r* per infection density of *H. schachtii*. The fitted line is from an exponential model, the *R*^2^ is from the fitted curve. (C) As in (A), but for infections with *M. incognita*. (D) As in (B), but for infections with M*. incognita*. The solid line indicates the tolerance limit. The dashed line indicates the confidence interval.

Pair-wise comparisons of green canopy areas between different treatments are cumbersome, and drawing conclusions remains elusive. Therefore, we fitted and tested our growth dynamics of green canopy areas (Figs 2–4; Supplementary Fig. S7) to a logistic growth model (Equation 3) to determine if Arabidopsis Col-0 is more tolerant to *M. incognita* or *H. schachtii*. The logistic growth model determined the maximum canopy area and the intrinsic rate of growth. We found that the maximum canopy area *K* had a relationship to *P*_i_ that could be fitted with a Gaussian curve (Equation 4) (Fig. 5A, C) and that the relationship between the intrinsic growth rate *r* and *P*_i_ could be fitted with a hyperbolic curve (Equation 5) (Fig. 5B, D). These fits allowed us to estimate the tolerance limits, which were *P*_i_=3.3 [95% confidence interval (CI) 3.0–3.7] for *H. schachtii* and 5.5 (95% CI 4.6–6.4) for *M. incognita*. We therefore conclude that *A. thaliana* Col-0 probably is more tolerant to biotic stress by *M. incognita* infection than *H. schachtii*.

## Discussion

Plants show intraspecific variation in how much damage by root-parasitic nematodes they can cope with before growth is delayed or ceases altogether (Potter and Dale, 1994). Unfortunately, uncovering the underlying molecular mechanisms of tolerance remains challenging and laborious since no tractable genetic model system is available. The goal of this research was 2-fold: (i) to assess the suitability of *A. thaliana* as a model for tolerance to root-parasitic nematodes and (ii) to develop a high-throughput phenotyping platform to quantify and measure tolerance levels. The suitability of *A. thaliana* to measure tolerance was demonstrated with the beet cyst nematode *H. schachtii* and the root-knot nematode *M. incognita*. We present a high-throughput phenotyping platform that allows for the collection of continuous plant growth data (here with a resolution of one data point per hour). Continuous data are an important improvement upon end point data which are normally used to determine tolerance levels of plants to nematode infection. Based on our findings, we believe that our phenotyping platform will enable us to uncover the underlying genetic mechanisms of tolerance.

We show that the green canopy area in *A. thaliana* is a suitable proxy to study tolerance limits and growth responses of plants to root-parasitic nematode infections in a high-throughput manner. Moreover, we were able to determine the tolerance limits of *A. thaliana* Col-0 to *M. incognita* and *H. schachtii.* The nematodes *H. schachtii* and *M. incognita* infect root tissues, but it is difficult and laborious to follow and quantify root development over time. Although we measured individual root traits (Fig. 1), the throughput and therefore the data density were low. Similarly, although the output of the number of flowers gives a similar trend to the green canopy area and could indicate similar or other tolerance mechanisms, the throughput of this parameter is low compared with green canopy area measurements. Previously, it was shown that green canopies are suitable to monitor the effect of nematode infection on sugar beet (Joalland *et al.*, 2016, 2017). Therefore, we monitored green canopy area growth of individual plants in a non-destructive way by taking pictures (Fig. 2). We showed that green canopy area is a good proxy for below-ground responses to nematode infection (Supplementary Fig. S3). Multiple root traits correlated well with the green canopy area. Furthermore, our approach allows capturing changes in growth rates in response to nematode infection over time (Figs 3, 4). By identifying dynamic damage responses and compensatory growth rates of nematode-infected Arabidopsis plants, we were able to determine their tolerance limits (Fig. 5). Even though we did not measure susceptibility (i.e. number of penetrations, feeding sites, or cysts/egg masses), tolerance is measured by correlating yield to the initial density of nematodes, not the reproductive rate (*P*_f_/*P*_i_) (Norshie *et al.*, 2011; Teklu *et al.*, 2022). Specifically, some susceptible plants (probably hosting more successful infections) are less affected than plants which are resistant (Evans and Franco, 1979). In other words, resistance and tolerance are genetically and physiologically different and independent traits. To our knowledge, this is the first time that tolerance limits are compared between nematode species.

We observed three distinct effects of infections by plant-parasitic nematodes on plant growth rate: an initial damage response; a growth rate recovery at early time points; and a growth compensation response at later time points. First, we observed that plants showed an initial damage response by slowing growth rates shortly after *M. incognita* inoculation (Supplementary Fig. S8). The growth rates recovered 4 d later. Similar observations were made for root growth upon prolonged periods of invasion by the cyst nematode *Heterodera avenae* (Rawsthorne and Hague, 1986). Secondly, we observed that *H. schachtii*-inoculated plants grew significantly more slowly compared with *M. incognita-*inoculated plants at 5–10 dpi under similar nematode inoculation densities (Supplementary Fig. S10). It is unclear whether the differential growth rates are due to differences in pathogenicity between the two nematode species, the types of damage that these two nematodes cause, or the interaction of the nematode species and Arabidopsis. Thirdly, at the later time points (16–21 dpi), plants inoculated with lower dosages of *H. schachtii* and *M. incognita* showed increased growth rates compared with mock-inoculated plants (Figs 3D, 4D). To our knowledge, this is the first observation of—in addition to growth recovery at early time points—growth compensation at later stages of infection. Yield losses by nematodes could be minimized if characteristics such as growth recovery and compensation were to be incorporated in breeding programmes. However, this requires more insight into the genetic (in)dependence of these growth–response traits.

At lower nematode densities, we observed enhanced growth in the infected *A. thaliana* Col-0 plants (Figs 1E, 2C, 5A, C). Earlier, we reported that at lower densities *A. thaliana* Col-0 root length also increases at lower densities (Guarneri *et al.*, 2023). Overcompensation, or hormesis, is a widely observed biological phenomenon, that has also been demonstrated in plants (reviewed in Agathokleous *et al.*, 2019). It has also been shown for plant diseases (e.g. Flores and Garzon, 2013; Di *et al.*, 2016). However, as far as we know, hormesis has not been reported in a quantitative relationship to disease pressure on plants, including in nematode infections. Rather, nematode infections are modelled using the SYLM framework (Sasanelli *et al.*, 2013; Been *et al.*, 2015; Moosavi, 2015). It is likely that the increased time resolution, the high level of experimental control, and the high number of densities measured by our platform allowed measurements of this effect. It is possible that a biotic hormetic effect can be specific to nematodes, as it is well established that the initial density of nematodes relates to the damage caused (Brown, 1969; Seinhorst, 1986). In many other pathogens (e.g. viruses, fungi, and bacteria) such dose–response relationships are not expected as these replicate multiple times within a growth season whereas nematodes are comparatively slow.

There seems to be a link between the infection stages of the nematodes and the changes that we measured in growth kinetics of host plants. The nematode infection process starts with root invasion, followed by migration through root tissue, after which feeding sites are formed, leading to loss of assimilates via these feeding sites. First, we believe that the slower growth rate during the first 4–5 d is due to *M. incognita* and *H. schachtii* invasion and migration (Supplementary Figs S6, S8). A faster recovery response by the plant to *M. incognita* infection than to *H. schachtii* infection could have four reasons: (i) *M. incognita* needs less time to induce a feeding site and therefore the plant can continue growth more rapidly; (ii) *M. incognita* causes less stress due to its different mode of invasion and migration; (iii) *M. incognita* is a slow-moving nematode compared with *H. schachtii*, which gives the plant more time to heal the associated damage caused during root invasion and migration; or (iv) the feeding site is induced at a different place in the root. Specifically, cyst nematodes can invade the root at different locations along the root axis, and subsequently migrate intracellularly through the cortex towards the vascular cylinder, whereas root-knot nematodes invade the root at the root tip and migrate intercellularly through the cortex and the apical meristem to enter the vascular cylinder from below (Wyss and Zunke, 1986; Wyss, 1992; Wyss *et al.*, 1992; Kyndt *et al.*, 2013). Finally, we measured a drop in growth speed, probably as a consequence of the loss of assimilates by nematodes feeding (reviewed in Trudgill, 1991; Grundler and Bockenhoff, 1997). After partial growth recovery in response to lower nematode densities for *M. incognita*- and *H. schachtii*-treated plants, we observed that growth rates slowed down again around 7 dpi (Supplementary Figs S6, S8). Mechanical or physiological damage during feeding site initiation and expansion, and consequently withdrawal of plant assimilates, are important factors that contribute to reducing plant growth and delaying development (Trudgill, 1991). Being able to measure the effects of different nematode life stages (invasion/migration and feeding) on plant growth could provide new and interesting insights into how plants respond to different types of stress (i.e. mechanical damage versus loss of assimilates). As we are able to phenotypically discriminate growth responses to different types of stresses, this indicates that tolerance is a complex trait. We hypothesize that tolerance is a combination of multiple (simultaneous) processes each with their own genetic architecture. Furthermore, it is possible that genotypic diversity in the root-parasitic nematodes can also play a role in the tolerance response. For instance, it has been reported for various species that populations differ in infectivity and virulence on different plants (Storelli *et al.*, 2021).

Our observation of the compensatory growth response shows that there are two limitations in the application of the SYLM on nematode-infected *A. thaliana*. First, the SYLM underestimates the tolerance limits for *H. schachtii* and *M. incognita* infections in Arabidopsis (Fig. 2C; Supplementary Fig. S7). Yet, the overall density response in our data fitted the model well. Similar observations were made previously where the tolerance limits strongly deviated between biological replicates (Teklu *et al.*, 2022). We conclude that although the SYLM does not perform well at low densities, it is suitable to predict minimal yields. Second, the SYLM is inefficient as it uses only end point data (Seinhorst, 1986; Norshie *et al.*, 2011; Sasanelli *et al.*, 2013; Been *et al.*, 2015; Moosavi, 2015), such as tuber yield or shoot biomass. The SYLM does not capture the complex growth dynamics. Insight into the complex growth dynamics is necessary to separate different tolerance mechanisms.

To conclude, plants display intraspecific growth responses to nematode infection. These differences in tolerance levels are an important agronomic trait of crops as they determine the level of stress that the plant can handle before growth is delayed. Due to the lack of a tractable genetic model system, the molecular mechanisms underlying tolerance remain elusive. Our high-throughput phenotyping platform which is capable of monitoring the effect of nematode infection on the growth of the model plant *A. thaliana* will provide novel insights into how plants mitigate the impact of damage by nematodes and how plants try to compensate growth delays caused by the loss of assimilate by feeding nematodes. Specifically, our high-throughput phenotyping platform will provide opportunities to identify novel insights into the molecular and genetic mechanisms that underlie tolerant growth responses, for instance by performing genome-wide association studies. Incorporating tolerance could provide an additional layer of protection for securing global food production in addition to disease resistance.

## Supplementary data

The following supplementary data are available at *JXB* online.

Fig. S1. Overview of the experimental setup.

Fig. S2. The primary root length and the green canopy area of Arabidopsis respond in a density-dependent manner to infection by *Heterodera schachtii*.

Fig. S3. Spearman correlation coefficients calculated between the average of different measurements of root components at 7 dpi and green canopy area at 11 dpi.

Fig. S4. Green canopy area of *Heterodera schachtii* inoculated Arabidopsis Col-0 plants measured over time.

Fig. S5. Calculated growth rate of Arabidopsis Col-0 plants inoculated with increasing densities of *Heterodera schachtii.*

Fig. S6. Growth rate of Arabidopsis Col-0 plants inoculated with increasing densities of *Heterodera schachtii.*

Fig. S7. The relationship between the inoculation density (*P*_i_) of *Meloidogyne incognita* and green canopy area (relative yield).

Fig. S8. Growth rate of Arabidopsis Col-0 plants inoculated with increasing densities of *Meloidogyne incognita.*

Fig. S9. Correlations between growth rates of Arabidopsis Col-0 plants inoculated with *Heterodera schachtii* and *Meloidogyne incognita* per dpi or inoculum density.

Fig. S10. Comparison of growth rates of Col-0 inoculated with *H. schachtii* and *M. incognita*.

Video S1. Growth of Arabidopsis Col-0 infected with *H. schachtii*.

erad266_suppl_Supplementary_Figures_S1-S10

erad266_suppl_Supplementary_Protocol_S1

erad266_suppl_Supplementary_Protocol_S2

erad266_suppl_Supplementary_Video_S1

## Data Availability

All relevant data within the manuscript can be found on Github (https://git.wur.nl/published_papers/willig_2023_camera-setup), or protocols.io (doi: dx.doi.org/10.17504/protocols.io.kqdg39167g25/v1). Also, the full picture dataset has been made available at doi: https://doi.org/10.6084/m9.figshare.23518923.v1. (Willig *et al.*, 2023)

## Abbreviations

J2s: infective second-stage juveniles
*P*_i_: nematode density upon inoculation
SYLM: Seinhorst yield loss model
