# Supplementary material for: From root to shoot: quantifying nematode tolerance in Arabidopsis thaliana by high-throughput phenotyping of plant development
Source: J Exp Bot. 2023 Jul 11;74(18):5487–99. doi: 10.1093/jxb/erad266 (PMC10540735; doi:10.1093/jxb/erad266)
Supplement: erad266_suppl_Supplementary_Figures_S1-S10 [file erad266_suppl_supplementary_figures_s1-s10.pdf]

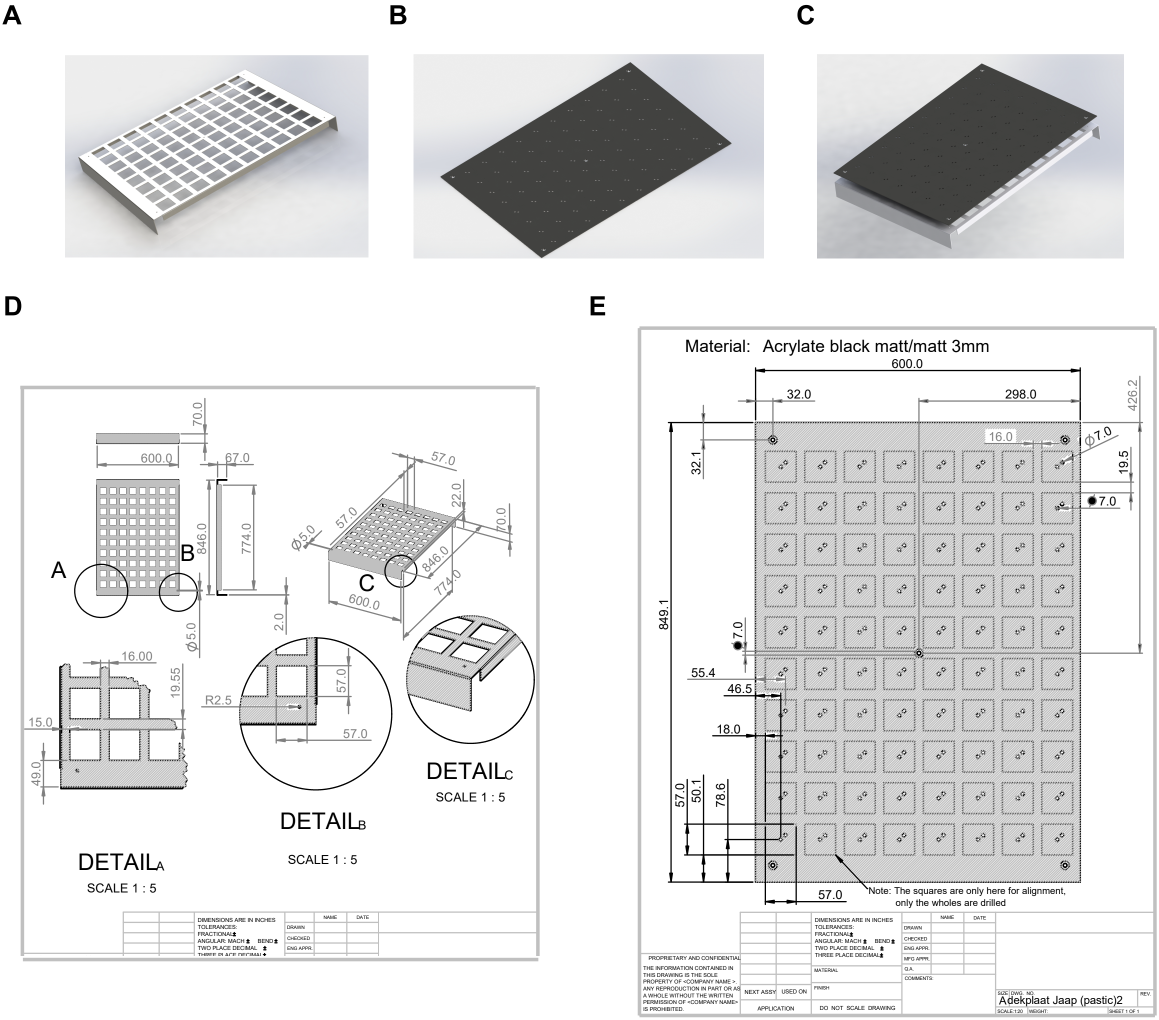

Figure S1: Overview of experimental setup. A and D) Stainless steel frames that we designed for holding pots. **B and E)** 3 mm thick black nonreflective foamed PVC coversheet drilled with countersunk holes and holes to inoculate nematodes. **C)** Black plates are attached to the aluminium frames with screws.

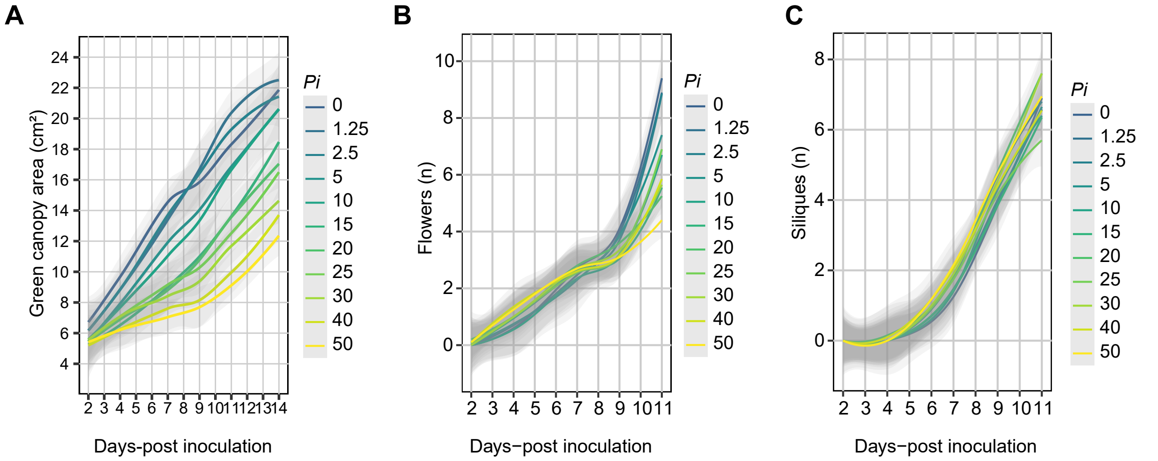

Figure S2: The primary root length and the green canopy area of Arabidopsis responds in a density-dependent manner to infection by *Heterodera schachtii*. Twenty-one-days-old Col-0 seedlings were inoculated with increasing densities of second-stage juveniles of *H. schachtii* (0-50 juveniles per g dry sand). At different timepoints, we counted the number of flowers and siliques (n=16-20). **A)** Effect of nematode inoculations on the green canopy over time. **B)** Effect of nematode inoculations on the number of flowers over time. **C)** Effect of nematode inoculations on the number of siliques over time.

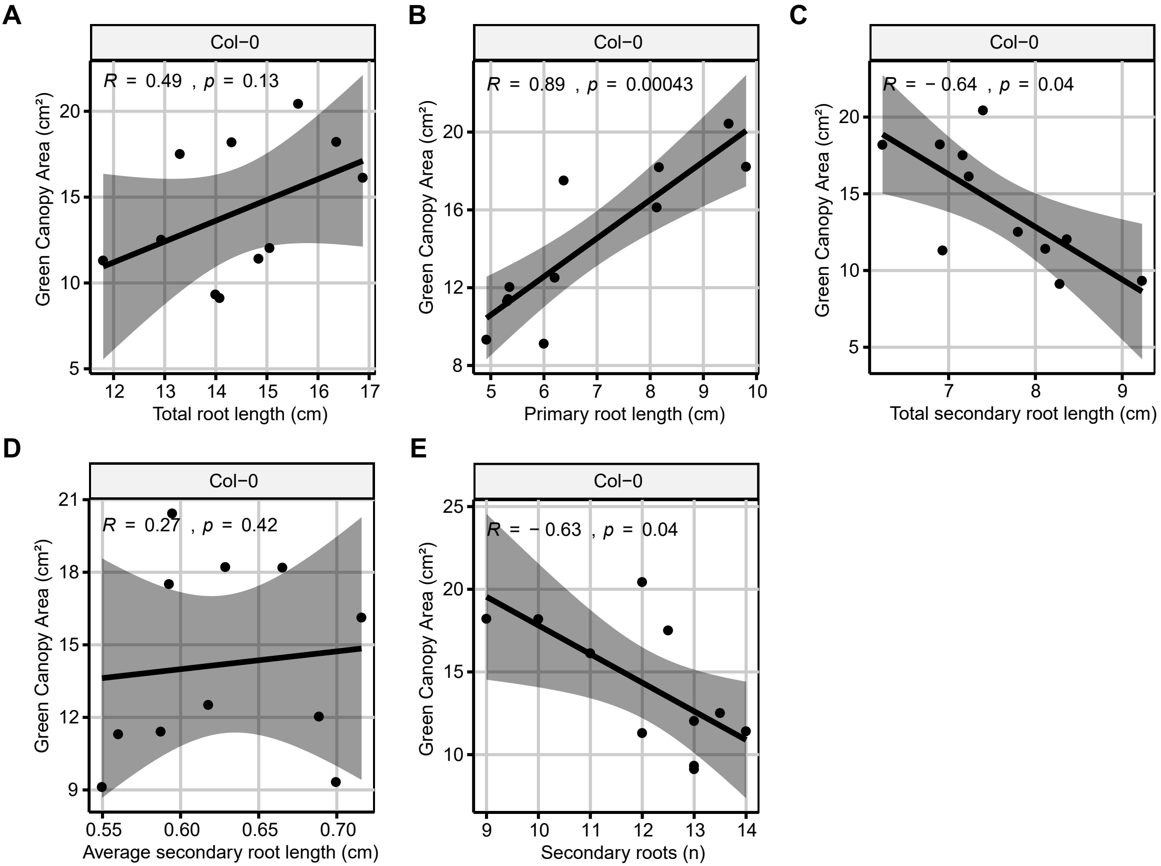

Figure S3: Spearman correlation coefficients calculated between the average of different measurements of root components at seven-days post inoculation and green canopy area at eleven-days post-inoculation. Spearman correlation coefficients were calculated on average values of 16-20 replicates of green canopy measurements and 24-30 replicates of root measurements using R software. **A)** Correlation between green canopy area and total root length. **B)** Correlation between green canopy area and primary root length. **C)** Correlation between green canopy area and total secondary root length. **D)** Correlation between green canopy area and average secondary root length. **E)** Correlation between green canopy area and the number of secondary roots.

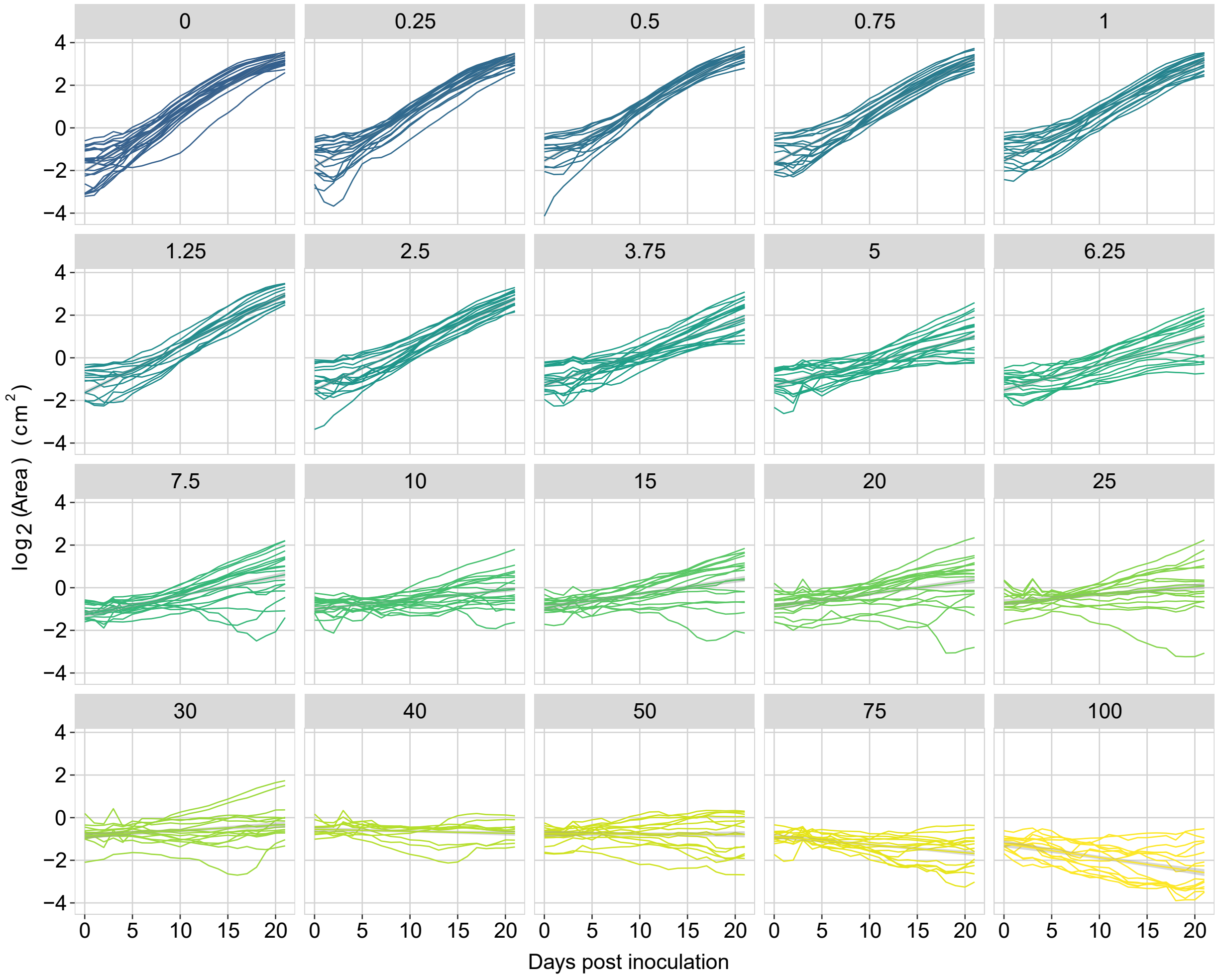

Figure S4: Growth of Arabidopsis Col-0 plants inoculated with increasing densities of *Heterodera schachtii.* Nine-day-old Arabidopsis seedlings were inoculated with 20 densities (P_i) of *H. schachtii* juveniles (0 to 100 juveniles per g dry sand). Lines represent the growth of individual plants

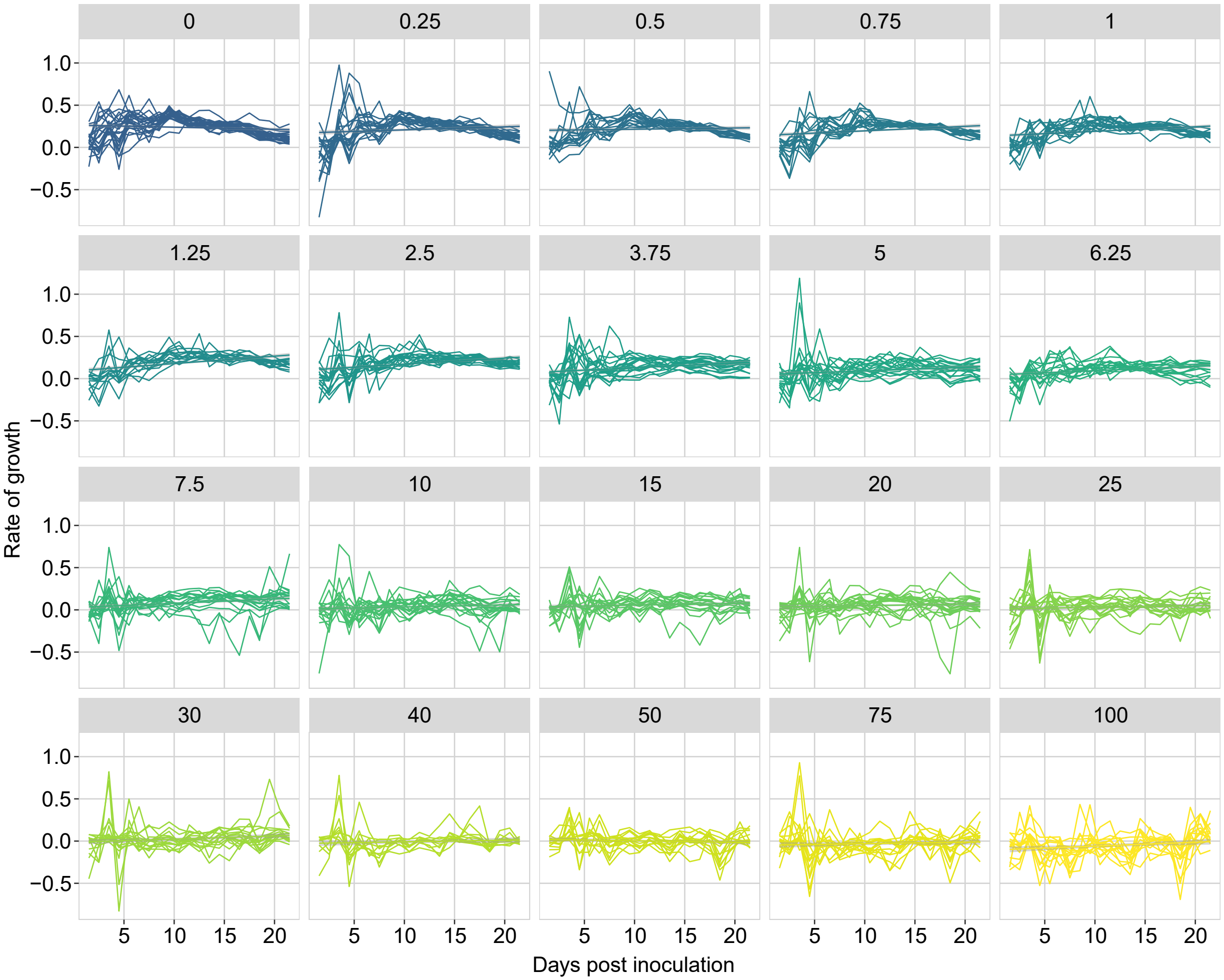

Figure S5: Growth rate of Arabidopsis Col-0 plants inoculated with increasing densities of *Heterodera schachtii.* Nine-day-old Arabidopsis seedlings were inoculated with 20 densities (P_i) of *H. schachtii* juveniles (0 to 100 juveniles per g dry sand). Lines represent the growth rates of individual plants

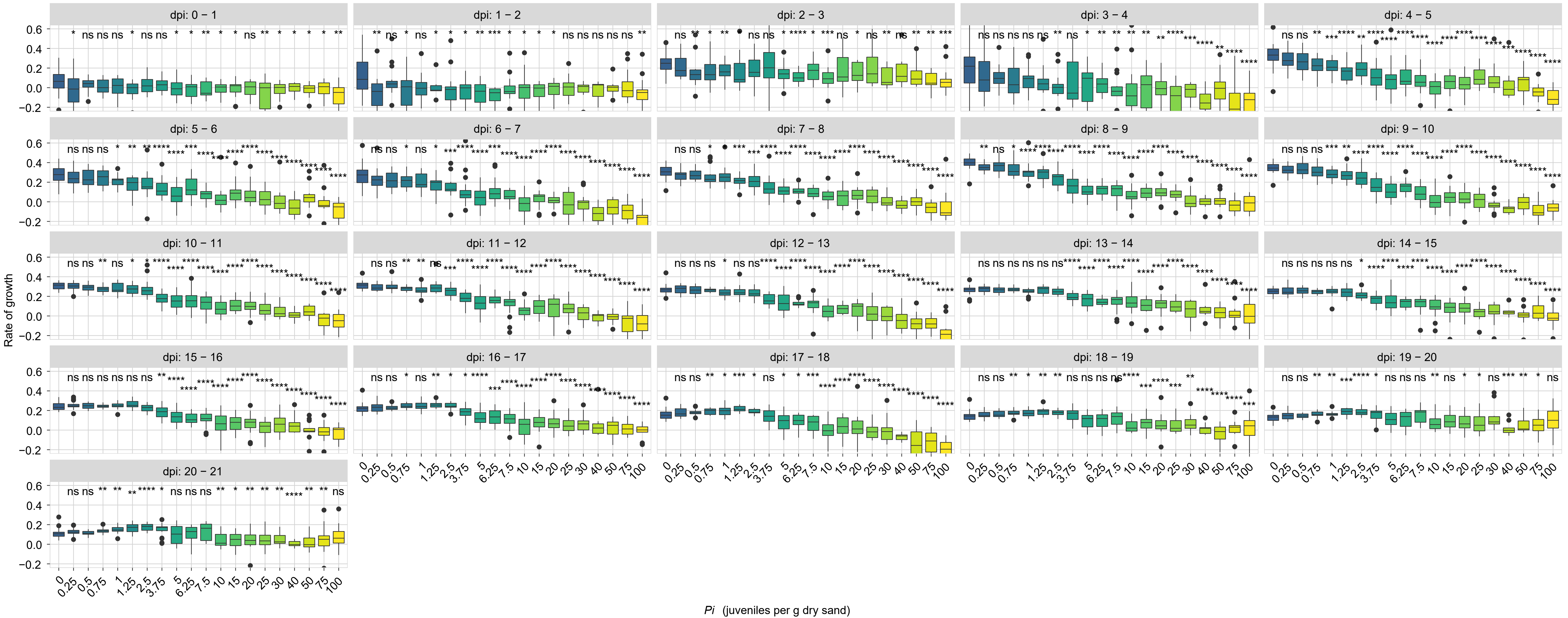

Figure S6: Growth rate of Arabidopsis Col-0 plants inoculated with increasing densities of Heterodera schachtii. Nine-day-old Arabidopsis seedlings were inoculated with 20 densities (*Pi*) of *H. schachtii* juveniles (0 to 100 juveniles per g dry sand). The growth rates of plants were calculated per day. Dots represent individual plants. Data was analysed with a Wilcoxon Rank Sum test. ns= not significant, *p< 0.05, **p< 0.01, ***p<0.001 (n=10-24).

Ecotype	*m*	*Te*	Ymax	se*m*	se*T*	se Ymax	*RSQ*	DF
Col-0	0.13	0.57	12.78	0.05	0.09	0.03	0.78	16

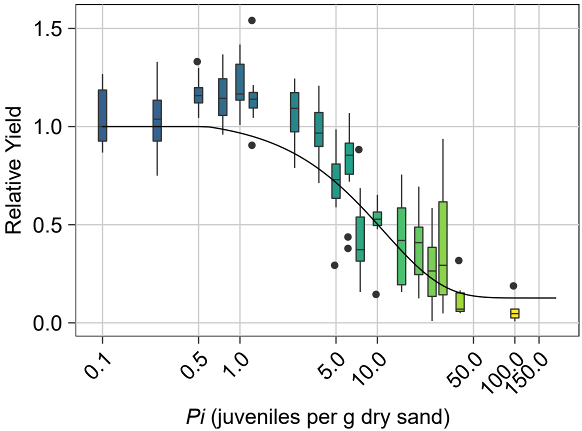

Figure S7: The relationship between the inoculation density (P_i) of *Meloidogyne incognita* and green canopy area (Relative Yield). Nine-day-old Arabidopsis seedlings were inoculated with 18 densities of *M. incognita* juveniles (0 to 100 juveniles per g dry sand). Line was fitted according to the Seinhorst yield loss equation: $y = m + (1 - m)\ 0.95^{Pi/T-1}$ for $Pi > T$ and $y = 1$ for $Pi \leq T$. Parameter values for Seinhorst's Eq. for the relation between initial population density (P_i) of *H. schachtii* and measured leaf surface area. P_i and tolerance limit (T) are expressed in *M. incognita* (g dry sand)$^{-1}$ while, the minimal yield (m) is the lowest proportion of the maximum green canopy area (cm^2) (Y_{max}) at 21dpi. The goodness of the fit is expressed in the *RSQ*.

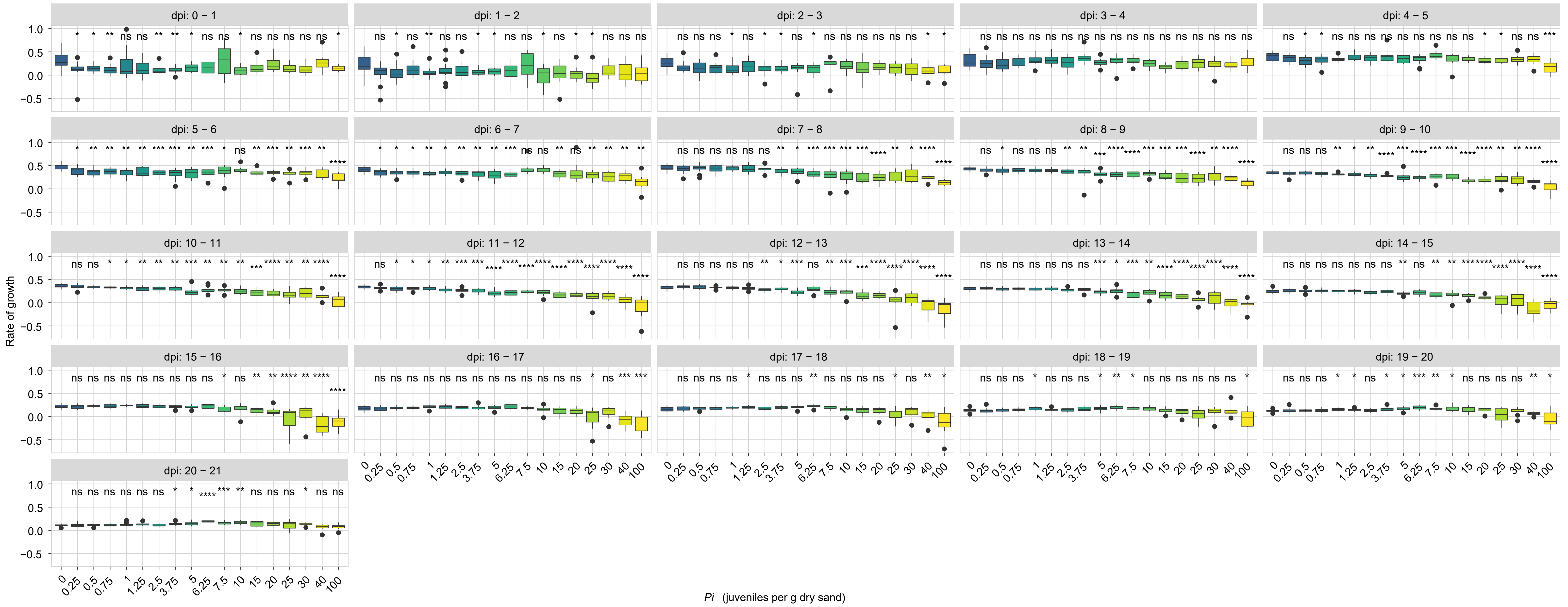

Figure S8: Growth rate of Arabidopsis Col-0 plants inoculated with increasing densities of *Meloidogyne incognita*. Nine-day-old Arabidopsis seedlings were inoculated with 20 densities (*Pi*) of *M. incognita* juveniles (0 to 100 juveniles per g dry sand). The growth rates of plants were calculated per day. Dots represent individual plants. Data was analysed with a Wilcoxon Rank Sum test. ns= not significant, *p< 0.05, **p< 0.01, ***p<0.001 (n=10-24).

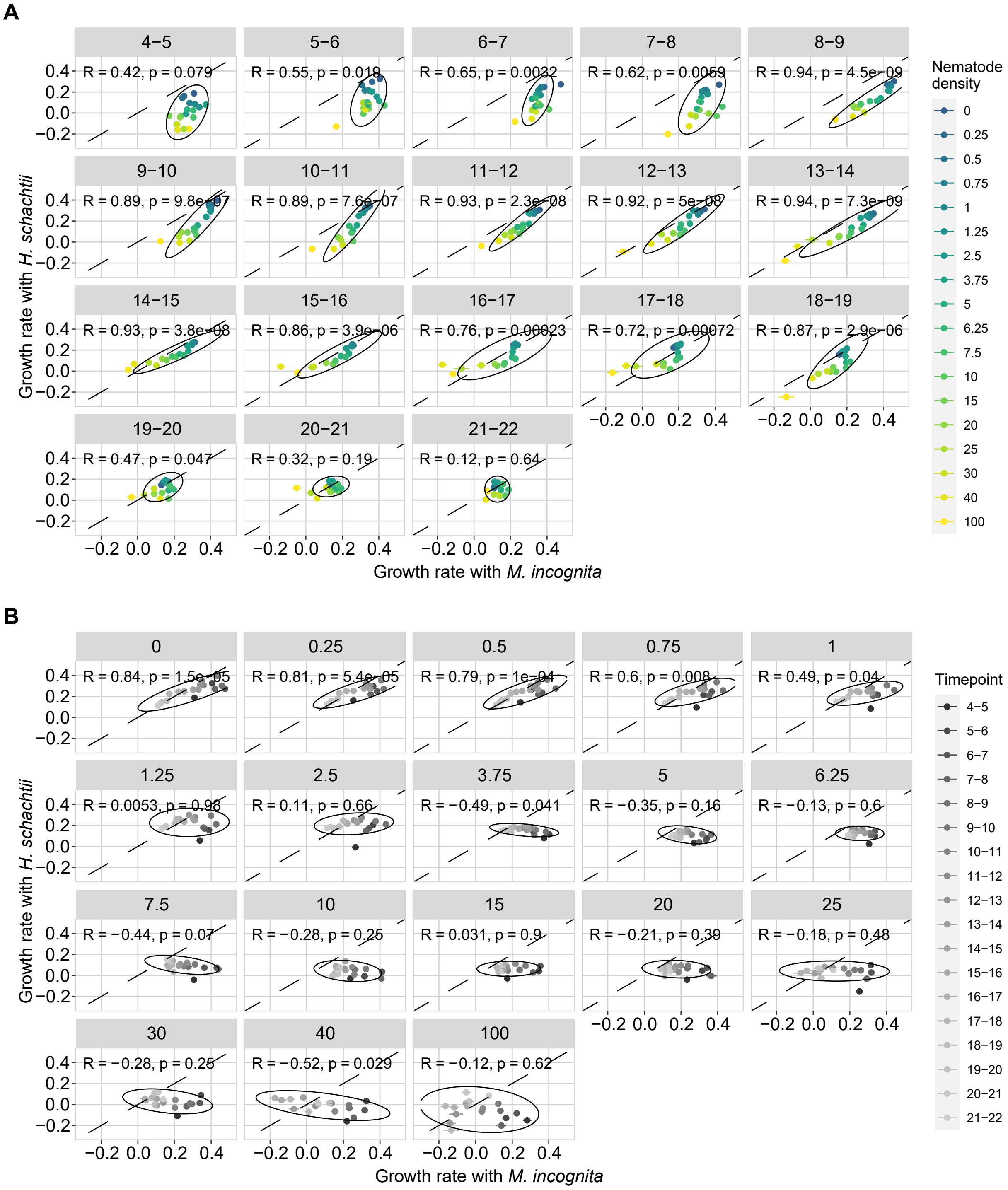

Figure S9: Correlations between growth rates of Arabidopsis Col-0 plants inoculated with *Heterodera schachtii* and *Meloidogyne incognita* per (A) dpi or (B) inoculum density. Nine-day-old Arabidopsis seedlings were inoculated with 18 densities (*Pi*) of *H. schachtii* and *M. incognita* juveniles (0 to 100 juveniles per g dry sand). The growth rates of plants were calculated per day. Dots represent the average growth rate. The correlation coefficient and p-values shown derive from a Pearson correlation

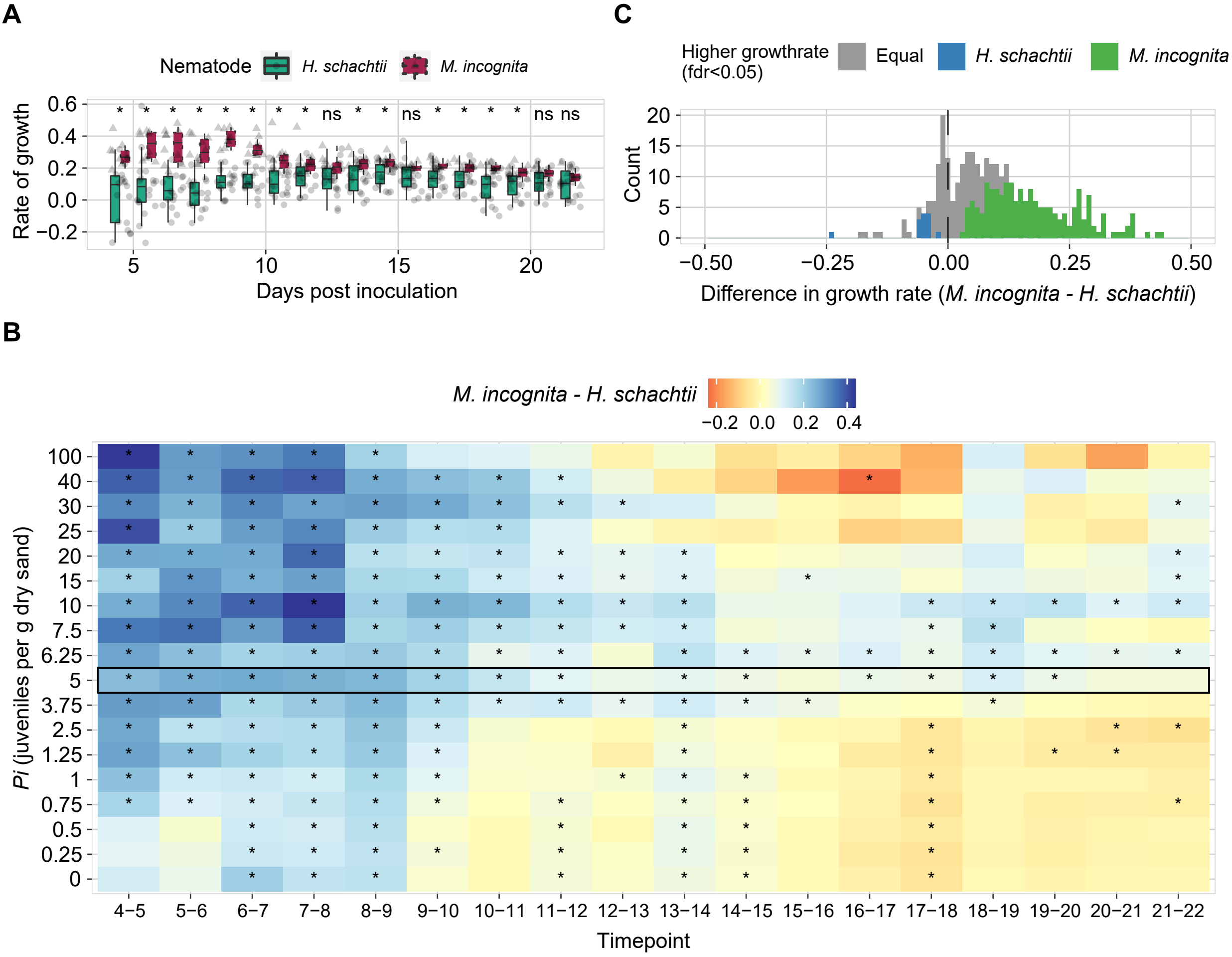

Figure S10: Comparison of growth rates of Col-0 inoculated with *H. schachtii* and *M. incognita*. Arabidopsis seedlings were inoculated with 18 densities (P_i) of *H. schachtii* juveniles or 18 P_i's of *M. incognita* (0 to 100 juveniles per g dry sand). Growth rates of green canopy areas were calculated from 0 to 22 dpi. **A)** Comparison of green canopy area growth rates of plants inoculated with P_i 5 of *H. schachtii* (full-line boxplots) and *M. incognita* (dashed-line boxplots) over time. **B)** Heatmap of the differential growth rate per P_i and timepoint. The difference in growth rate was calculated by subtracting the *H. schachtii* treated growth rates from the *M. incognita* treated growth rates per timepoint and P_i, blue indicates that *M. incognita* inoculated plants have a higher growth rate than *H. schachtii* inoculated plants and red indicates vice versa. **C)** Histogram of the number of occurrences that growth rates of *H. schachtii* and *M. incognita* inoculated plants significantly differ per P_i and timepoint. Blue represents significantly higher growth rates for *H. schachtii* inoculated plants, grey indicates no significant differences, and green represents significantly higher growth rates for *M. incognita* inoculated plants. Differences in growth rate between plants infected with either *H. schachtii* or *M. incognita* were tested using a paired t-test comparing data from the same day and density combination; ns= not significant, *p< 0.05 (n=10-24).
